# Towards a Dignified Death: A New Approach to Care for People Using Substances Who Are at, or Near, the End of Their Lives

**DOI:** 10.3390/ijerph20105858

**Published:** 2023-05-18

**Authors:** Sarah Galvani, Sam Wright, Amanda Clayson

**Affiliations:** 1 Sociology Department, Faculty of Arts and Humanities, Manchester Metropolitan University, Manchester M15 6LL, UK; samantha.wright@mmu.ac.uk; 2 VoiceBox Inc., Bolton, Lancashire BL2 1DW, UK; amanda@voiceboxinc.co.uk

**Keywords:** dying, death, palliative, alcohol, drugs, theory of change, model of care, practice

## Abstract

There are no effective intervention studies for people using substances who are at, or near, the end of their lives. The needs of this group of people have been consistently overlooked even within the literature that identifies marginalised groups of people in need of greater recognition in palliative and end-of-life care. The aims of the project were to: (i) determine what a new, co-produced, model of care should look like for people using substances needing palliative and end-of-life care, and (ii) establish whether the new model had the potential to improve people’s access to, and experience of, end-of-life care. This paper presents the development of the new approach to care. It was developed using participatory action research principles over a course of online workshops during the COVID-19 pandemic lockdown period in the UK. A theory of change that aims to inform future policy and practice development is presented. While the ambition of the research was stunted by the pandemic, the process of its development and dissemination of the model and its resources has continued. Response from participants highlighted the importance of this work, however, in this new field of policy and practice, preparatory work that engages a wide range of stakeholders is crucial to its success. This relationship building and topic engagement are major parts of implementation before more substantial and sustainable development goals can be met.

## 1. Introduction

The marginalisation from mainstream society of people who use illicit substances either recreationally or chronically is not new. This extends to people who use legal drugs, such as alcohol or prescription drugs, but only when their use is considered excessive and detrimental to their own health and wellbeing, or to the health and wellbeing of those around them. In the United Kingdom (UK), deaths from alcohol and other drug use are at the highest they have ever been [1,2]. This follows more than a decade of funding cuts for the services that support people needing help [3]. At the same time, and in common with most of the world’s population, the UK’s population is ageing [4], with much larger groups of people reaching older age and reaching it with more complex health and social care needs. United Kingdom data show this ageing is reflected in the profile of people most likely to have alcohol-related hospital admissions [5] and drug-related poisoning and deaths [2].

Trends of people in treatment for substance use in the UK also show an increase among older age groups [6]. These data underpinned our research hypothesis that people who use substances are likely to be increasingly visible within palliative and end-of-life services and that practitioners in substance use services are more likely to be working with older people with a range of life limiting health and care needs. Palliative care is care for people who are terminally ill and their families. It seeks to maximise their physical health and their emotional, social, and spiritual wellbeing and provide the best quality of life possible in the period leading to a person’s death [7]. End-of-life care definitions vary. It is often considered to be the last 12 months of someone’s life, but it can mean the last days and weeks of a person’s life, given that the end of life is difficult to determine [8].

Subsequent consultation with a front-line substance use service in the Midlands and a North-West hospice suggested a notable increase in people presenting to their services with terminal health conditions and ongoing chronic use of alcohol or other drugs (hereafter, substance use). The substance use service reported people with advanced liver disease entering their service in the hope of improving their health and wellbeing [9]. Hospice staff reported seeing more people who were engaged in substance use—both inpatients and outpatients. Both service providers felt they needed to know more and do more for this group of people and improve their policy and practice [10]. As a result, these two services and five others became partners in a six-strand exploratory research project to identify current good practice interventions for people using substances approaching end of life [11]. The study found that there were no effective interventions identified in the existing literature [12] and that people with lived experience of substance use and serious ill health often received limited care accompanied by stigmatised care responses [13]. Further, they and their families identified a number of missed opportunities for supportive professional intervention [14]. Family members (including friends and unpaid carers) had no formal support from health and social care agencies despite experiencing significant negative impact from living with, or caring for, someone using substances approaching end-of-life [14]. These findings led to a subsequent research project, funded by the National Institute for Health Research in the UK, that aimed to develop and disseminate a new model of care for this group of people.

This paper sets out the early stages of the model development, in particular the co-construction of a theory of change and the resultant logic model, hereafter known as the model of care (MoC). This is the first project of its kind internationally, and it is, therefore, important to share the lessons learned in developing and disseminating the model and the need to readjust and manage expectations in this new area of research and practice.

## 2. Methodology

The aims of the project were to (i) determine what a co-produced model of care should look like for people using substances needing palliative and end-of-life care, and (ii) establish whether the model had the potential to improve people’s access to, and experience of, end-of-life care. One of its main objectives was to develop and implement a co-produced, integrated model of care in partnership with people with lived experience of substance use (current or past), and serious and advancing ill health, and with their families, friends, carers (FFCs), and professionals.

The focus of this paper is on the development of a theory of change to underpin the new logic model or model of care.

### 2.1. Methodology

The dominant methodology for this project was Participatory Action Research (PAR). This comprises a partnership approach to research involving people with different areas of expertise. Pain et al. [15] suggests PAR is used when care improvements and changes are required, indicating a good fit with the project’s aims. PAR assumes that the project is driven by all partners, not solely the research team. In this context, this research team is comprised of academics, practitioners, and people with lived experience. This can be a challenging approach for academics who are used to holding the power and controlling the project’s direction, as well as a challenge to the academic ‘rules’ and procedures, for example, ethical considerations, that surround research practice [16]. It can also be challenging for funders, as they want to know exactly what will be performed before committing to financing a project, whereas PAR requires activity to be determined through ongoing collaboration. The academic team comprised colleagues from a range of disciplines, including sociology, nursing, social care, and social work. These colleagues had worked on the previous project that underpinned this research, bringing with them extensive knowledge of the topic. The practice partners were approached by the research team as key statutory or voluntary sector service providers in Liverpool and Sefton, whose specialist expertise focused on one of the following: substance use, palliative and end-of-life care, housing support/homelessness, and adult social care. The participants with lived experience were recruited from the extensive community networks developed by our co-applicant with lived experience. They were people who were using, or had previously been using, substances that resulted in problems in their lives and their own or others’ ill health. They also included FFCs of people who died as a result of terminal illness caused, or exacerbated, by their use of substances. They were invited to take part once their experience of substance use and serious and advancing ill health had been established. Participatory action research was a good fit for the process involved in the development of the model, given that the first step was to construct a theory of change (ToC) to underpin it. According to Assmusen et al. [17] (p. 16), the “core purpose” of a ToC is “to specify *why* the intervention’s outcome is important” compared to “*how* an intervention will achieve its primary outcomes” in a logic model (*emphases in original*). There is no single format for developing ToCs [18], but, given our PAR approach, collaborative workshops [19] were considered the most appropriate method.

For the development of the theory of change, we facilitated participatory workshops. Workshops in research are designed to involve small numbers of people to ensure everyone can participate actively, thereby influencing the direction the work takes resulting in a new outcome [20].

### 2.2. Workshop Participants: Profile

The range of personal and professional expertise among our partners encompassed palliative and end-of-life care, substance use, mental health, and homelessness. People with lived experience included people who were ill and/or were using substances or had performed so in the past, as well as family members. Practice partners came from a mix of disciplinary backgrounds within health and social care and from a range of positions held within those agencies, including social workers, homeless support staff, GPs, drug and alcohol workers, service managers, and medical directors.

Four members of the project’s PEAT (People with Experience Advisory Team) were involved in the workshops alongside frontline representatives from the project’s 10 partner agencies, as well as three research team members. Representatives from the agencies self-selected to join the workshops. People were asked to attend two half-day online workshops, either workshop one or two, and either workshop three or four. This was to maximise attendance, given the ongoing pandemic and work pressures.

The geographical location for the project was a major city in the North-West of England (hereafter, the City). Prior to the start of this project, leaders from key substance use, end of life, and other social and health care services in the City, had been meeting to discuss the increasing number of people they saw who were using substances and approaching the end of their lives. The leaders reported limited, if any, health or social care for this group of people. These professionals became key supporters of this research through involvement as partner agencies or as members of its project advisory group.

### 2.3. Data Collection

COVID-19 restrictions in the UK meant the participatory workshops were held online using Microsoft Teams software, as meeting in person was prohibited by the UK Government. Moving the workshops online facilitated the model’s development due to reduced time demands on participants, but it may well have excluded those without access to the internet and/or the appropriate technology.

The four participatory workshops were run with different goals. Workshops one and two clarified what a theory of change was, its purpose, and began constructing the long-term goal for this project. Workshops three and four focussed on the model and developing medium and short-term goals, converting the theory of change into a more action-oriented logic model [17] or model of care. To develop the ToC, the discussion in workshops one and two was structured around what the group wanted a new model to achieve in the long term and why. The discussion was broad-ranging and centred on criticisms of existing provision, gaps in services, and who needed to do what to improve care. These workshops comprised full group and small group discussions to ensure all voices and views were heard. Workshops three and four added detail and activities to develop the model of care, with the goal of reaching agreement on the medium-term and short-term outcomes. The workshops also discussed what obstacles might need to be changed, and it reached the identified outcomes.

The workshops were recorded by handwritten or field notes taken by three research team members. Field notes are a data collection tool used in PAR [21], particularly where group discussion can make accurate voice recording and transcription more difficult. Three researchers, including our people with experience lead, took notes as a quality control mechanism to ensure that all contributions were noted. It also acknowledged that different subjectivities can affect what is heard.

### 2.4. Data Analysis

Data analysis involved reducing the field notes into a smaller number of categories without minimising the broader discussion. This was performed by a collaborative coding approach within the research team, talking through the key messages we heard, discussing overarching themes and topic clusters, agreeing key outcomes and activities, checking with workshop participants, then fitting these categories to a standardised template for a theory of change and logic model [17].

The draft theory of change was disseminated via email to workshop participants for comments and refinement, ensuring the views of all participants were adequately represented. The key skill for the research team was to distil the views, experiences, and suggestions of the workshop participants into the summative descriptions required in the ToC and MoC without excluding people’s views. This was achieved through a process of summarising and checking with participants during the workshop then inviting comments on the draft ToC. The resulting ToC and MoC are presented below.

Ethical approval for the research was gained through the UK’s Health Research Authority approval process (REC reference 20/WM/0140). Additional approval was required by some of the 10 partner agencies. In total, ethical approval processes took eight months.

## 3. Findings

**Figure 1:**
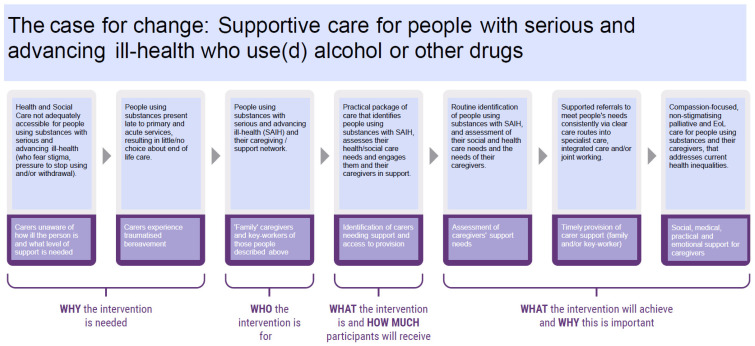
The Case for Change.

The agreed long-term goal of a new intervention was to achieve ‘compassion-focused, non-stigmatising palliative and end-of-life care for people using substances and their caregivers, that addresses current health inequalities’ (see Figure 1 below). This was reflective of participants’ experiences that current care was not accessible for this group of people who faced stigma and pressure to stop using substances if they did. This resulted in people attending primary and acute services at such a late stage of ill health that they had little or no choice about their end-of-life care. What participants wanted was for practitioners to have practical resources that help them to identify and assess people’s needs sooner, including their wider social and health care needs. This would be followed by supported referrals to meet those needs with clear routes into care and joint working once there.

The participants with lived experience raised the question about language and terminology and whether it could be changed to make it more accessible. As a result, the Theory of Change became the Case for Change. The language of palliative and end-of-life care was changed to ‘serious and advancing ill health’, as palliative is not a commonly used term outside of specialist services, and many people would not understand it. In addition, the subsequent model of care needed to reach across all disciplines and partner agencies. We heard that identifying people who needed palliative or end-of-life care may lead some practitioners to discount people they were supporting through a lack of a formal palliative or end-of-life diagnosis. Changing the terminology to ‘serious and advancing ill health’ would encourage practitioners to think more widely about the people in their services who they knew were unwell and may need additional support. Additionally, for people with lived experience and family members, it was a less fearful term than ‘end of life’ and may enable them to consider whether their own health, or that of their friend or relative, was deteriorating.

An additional layer was added to the Case for Change by the research team in consultation with the workshop participants to recognise and promote the needs of families, friends, and carers of people using substances and approaching the end of life. FFCs are a group of people who can experience considerable trauma and negative effects of a relative’s substance use and palliative care or end-of-life needs. They need support, in their own right, during the palliative phase and after the loss of their relative. For families, the goal was to have a range of support to help them in their caregiving roles, reflecting the absence of any support at present. The original research revealed how carers were often unaware of how ill the person was, resulting in their own traumatised bereavement when the person died [14]. The key service gaps highlighted were the identification of carers needing support through accessible assessment processes and the subsequent provision of carer support.

**Figure 2:**
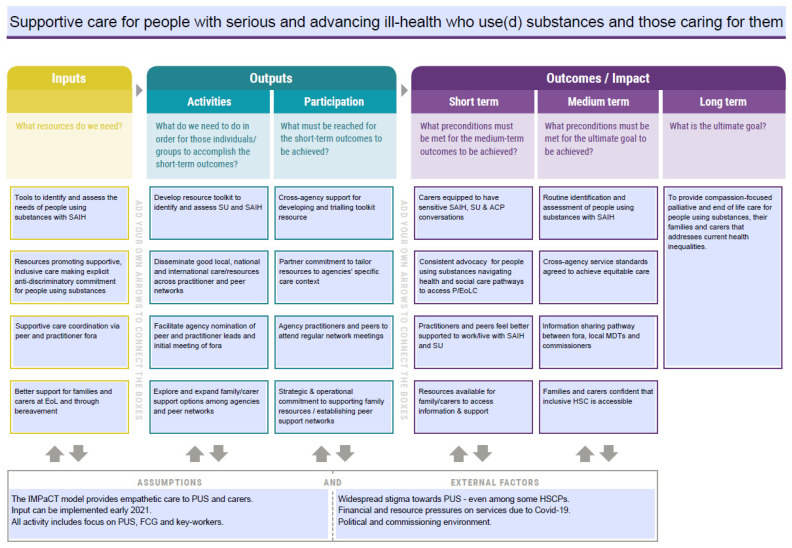
The Model of Care (Abbreviation list: SAIH—serious and advancing ill health; SU—substance use; EoL—end of life; ACP—advance care plan; P/EoL—palliative and end of life; MDT—multi-disciplinary team; HSC—health and social care; HSCP—health and social care professionals; PUS—people using substances; FCG—family care givers).

Participants shared many experiences of negative or absent care for people using substances near the end of their lives. Practitioners described feeling ignorant and unsupported, as well as not knowing who to ask when they needed specialist help. Participants also had a long list of desired outcomes, including improved knowledge and resources and networking that needed to be respectfully distilled into an achievable and focussed number of outcomes. Figure 2 (below) shows the final model of care. While workshops 1 and 2 provided adequate time to develop the theory of change, the two half-day workshops focusing on the detail and activities (workshops 3 and 4) required more time than anticipated to discuss, document, amend, and finalise the model’s detail. To avoid rushing the model, the research team made additional contact with the practitioners and the lived experience partners via email for feedback and refinement of the model. Some practitioners did not respond—possibly as a result of COVID-19-related work pressures—but feedback was received from all other participants. Additionally, one policy maker, who had been unable to attend the workshops, also responded.

The goal in Figure 2 replicates the goal in the Case for Change. The medium- and short-term goals were developed by exploring the links between the various gaps, requests, wants, and hopes identified in the workshops. This exploration and organising of the data continued until we found a clear backwards pathway from the long-term goal to the ‘inputs’ needed to begin the process of achieving it. The final four strands of the model reflected the tools, resources, and support identified as lacking and needing to be developed. These sit alongside their associated activities and the requirements for partner participation to work towards the short-, medium-, and long-term goals; these are goals that reflect values of compassionate and non-judgemental care, collaborative and informed practice, and improved service access.

The research team focussed on trying to achieve the short-term outcomes, given the limited duration of the project, as well as developing the ‘inputs’ and ‘activities’ that would contribute to meeting them. The activities required the development of tools to help people (i) identify and assess substance use and serious and advancing ill health, (ii) disseminate good local, national and international care/resources across practitioner and peer networks; (iii) facilitate agency nomination of peer and practitioner leads and meeting of initial fora; and (iv) explore and expand family/carer support options among agencies and peer networks.

In total, 17 written outputs were developed. These comprised information posters, practice guidance, information booklets, practice pointers, and family-focussed literature. In addition, two recorded podcasts of discussion between four experts in homelessness, substance use, and end-of-life care resulted in over 40 podcast segments for use in teaching and learning. A website was developed to host these resources focussing on three groups: (i) individuals living with substance use and serious and advancing ill health, (ii) family, friends and carers, and (iii) practitioners [22]. In addition, the research team facilitated support groups for practitioners and family members, not people with lived experience (PWE), as originally intended. Practitioners advised that there was already some support for PWEs, but nothing for family members, and this should be our support group focus. Six support forums were held. The three practitioner support groups attracted 20–25 people each time and seemed popular and helpful. The three family groups were not very large, with only one family member outside of the research team attending one of the three groups, despite offering a range of times and media by which to join and also advertising well in advance.

Training in the new model, how it had been developed, and the new resources was completed with 164 people across 11 health and social care providers. There was also a dissemination event at a café venue in the City with 20 community ‘connectors’, a group of people with lived experience whose reach spanned peer networks, key care agencies, and their own employers, who could disseminate the work and resources further.

The responses to, and feedback on, the training and the model were positive. However, they reflected a workforce who had not considered this issue before, for example, comments included “thought provoking” and “enlightening”, “really helpful and informative”, and “really interesting and useful”. There were some exceptions where the feedback indicated that more was going to be done within their organisations, for example, “I look forward to seeing where we can go with this work” and the resources had “already been shared with colleagues across the hospice.”

It was clear to the research team that what had been achieved with most agency staff, and the community connectors, was good initial engagement in an area of practice they had not previously considered. In addition, the project had clearly raised awareness, rather than effecting the more ambitious, and intended, practice change. One hospice and one housing agency were clearly exceptions, where additional resources had been allocated to working specifically with people who were using substances and who were homeless.

## 4. Discussion

Attempting this complex project was going to bring challenges. This was an ambitious project, even without a global pandemic impacting heavily on the involvement of our two core groups of experts, (i) social and health care providers and (ii) people with lived experience [23,24]. The research team was working with 10 organisations, only two of which had previously started work across the two fields of substance use and palliative or end-of-life care. The goal of practice change, therefore, was a huge task. Further, the lack of senior policy involvement was a disappointing gap in the team developing the model and raises important questions about what will happen beyond the research without their support to help drive it forward and to resource the activities required to reach the long-term goal. Despite initial willingness to participate, the regional policy makers were notably absent from the ToC and MoC workshops. This was an early concern given that policy maker involvement is an important component of successful implementation of new models of care [25]. This absence was thought to be indicative of a political crisis in the City at the time, compounded by the demands placed on senior health and social care staff during the COVID-19 pandemic.

The research team collaborated with many individuals who committed substantial amounts of time to the project, but implementing change requires a level of strategic action from policy makers, senior managers, and operational staff. This appeared absent in many cases, despite early promise. It is possible that there was an emotional element to it, as addressing death and dying can be painful for practitioners in the substance use field who went into careers to help people ‘recover’ from substance use. There may be an equally painful awareness among palliative and end-of-life care staff of the tensions between wanting to provide a dignified end of life and the life circumstances of people dependent on substances. Such unmanageable variables highlight the reality of conducting a study of this kind across front-line health and social care sectors and how the reality of development and implementation of new models of care in a real-world context is often ‘messy’, difficult and derailed.

The outcomes from the participatory workshops reflected the gaps in knowledge, resources, and service provision. They also reflected the ambition of the working group to improve the service offered to people using substances approaching the end of their lives. This created a tension between creating a model that was achievable and would maximise its chances of ‘successful’ implementation or documenting expressed need and delivering only part of it. The principles of participatory action research place the voices, views, and experience of participants as central to the research [26]. Therefore, documenting the ambition, while not achievable within the project timeframe or resources, was vital to ensuring their involvement was accurately reflected in the model.

The dissemination and training events highlighted how, with some exceptions, the research team may have had overly ambitious hopes for the implementation of this model at an agency level. The evidence reviews and the current research demonstrated the gaps in, and need for, improved service provision and a new approach to care for people using substances near the end of their lives. This, combined with our supportive contact with senior management in partner agencies, had created an assumption that staff in services would be ready to take this on. While we had “explicit buy-in” [27] for the work from key partners, the challenging socio-political environment of austerity and imminent recession [28], in addition to the pandemic’s impact on staff and services, meant staff were highly stretched. In their 14-step Quality Implementation Framework, Meyers et al. [28] stressed the importance of organisational readiness and capacity. It was clear from front-line staff responses to the training and dissemination events that mental and physical capacity to consider something new remained a challenge.

It is also possible that the research team’s immersion in the topic had arguably led to its unconscious competence, whereby the researchers did not realise the extent of their knowledge on this topic and thought the agencies and staff would be further on in their thinking and practice development than they were.

It was clear that much more time was needed to be built into the pre-implementation process to allow ideas and new information to settle, time to reflect and consider its relevance in relation to the people they supported, time to think about fit with current practice, time to look at the developed resources, and time to discuss with colleagues. Only then could the model be reviewed, revisited, and embedded into each agency’s practice, procedures, and policies, as well as tried out in practice.

Pain et al. [15] might refer to this as the reflection stage following, and preceding, an action stage in participatory action research. In a new area of research and practice, however, with new models of care to disseminate and implement across health and social care, more than reflection is needed. This is particularly important where new topics can challenge the status quo and where they may not previously have been considered. What is required between action and reflection is a period of *active engagement*, requiring ongoing support from the research team to further develop knowledge and support policy and practice change.

## 5. Conclusions

This paper reports on a new area of research and practice—the co-construction of a model of care for people in need of palliative and end-of-life care who are using substances. The development of the theory of change and a subsequent model of care offered an opportunity to work in partnership with a combined group of people with lived experience and practice agencies. It enabled their voices to be central to the development of the model. The outcome was an ambitious programme of change with long-term goals requiring additional resources and system change. The successes of this research lie in the engagement of practitioners and community connectors with this topic and the development, and dissemination, of resources to support knowledge acquisition. The challenges lie in its application to practice and the move from information generation to changing practice and procedures, despite organisational buy-in from the outset. Additional time is needed for active engagement, a stage between reflection and action, which enables practitioners and community connectors to consider the implications for their work and to ‘try out’ any implied changes to their care approach. Ultimately, it is the embedding of the activities within the MoC that will begin to address the gaps in service provision and the limited to poor levels of care experienced by people using substances approaching the end of their lives. However, this requires knowledge acquisition and active engagement with the topic by all professionals, followed by improved interagency working, improved practice with individuals and FFCs, and a holistic service provision that offers people using substances dignity at the end of life.

## Data Availability

Data sharing is not available for this article.
